# Occurrence, Distribution Characteristics, Risk Assessment, and Climatic Drivers of Type B Trichothecenes and Their Transformation Products in Major Wheat-Producing Areas of China

**DOI:** 10.3390/toxins18030150

**Published:** 2026-03-21

**Authors:** Jie Wang, Yu Wu, Di Cai, Li Li, Songshan Wang, Yu Zhang, Xiaomin Han, Songxue Wang, Leiqing Pan, Jin Ye

**Affiliations:** 1 College of Food Science and Technology, Nanjing Agricultural University, Nanjing 210095, China; 2023808109@stu.njau.edu.cn; 2 NFSRA Key Laboratory of Grain and Oil Quality and Safety, Academy of National Food and Strategic Reserves Administration, Beijing 100037, China; wyu@ags.ac.cn (Y.W.); cd@ags.ac.cn (D.C.); ll@ags.ac.cn (L.L.); wss@ags.ac.cn (S.W.); csuftzy@163.com (Y.Z.); wsx@ags.ac.cn (S.W.); 3 College of Food Science and Engineering, Central South University of Forestry and Technology, Changsha 410004, China; 4 NHC Key Laboratory of Food Safety Risk Assessment, China National Center for Food Safety Risk Assessment, Beijing 100021, China; 5 School of Public Health, Southern Medical University, Guangzhou 510515, China

**Keywords:** type B trichothecenes, deoxynivalenol, deoxynivalenol-3-glucoside, nivalenol, dietary exposure assessment, watershed-based analysis, climatic drivers

## Abstract

Type B trichothecenes (B-TCTs), predominant mycotoxins in wheat, threaten human health. However, their contamination profile in China, a major wheat producer, remains unclear. This study analyzed 1337 wheat samples (2022–2024) from main production areas using liquid chromatography-mass spectrometry and deterministic assessment to investigate B-TCTs’ watershed-scale distribution, spatiotemporal variations, associated health risks, and key climatic drivers. Results indicate that deoxynivalenol (DON) and its transformation product DON-3-glucoside (DON-3G) were the predominant contaminants, while nivalenol (NIV) was detected in specific river basins. Although overall exposure was low, elevated risks were identified in certain basins during particular years, especially for young children. DON-3G contributed 23.5% to total DON exposure. Relative humidity (rs = 0.34, *p* < 0.01), precipitation (rs = 0.37, *p* < 0.01), and its duration (rs = 0.38, *p* < 0.01) during the flowering-to-harvest period were identified as critical climatic drivers. The findings highlight the need to include DON-3G in food safety regulations and to develop climate-adapted control strategies.

## 1. Introduction

Trichothecenes (TCTs), which are sesquiterpene mycotoxins produced by *Fusarium* species and classified into four structural groups (A–D), contain an epoxy functional group that defines their toxicity [1,2,3]. Among these, type B trichothecenes (B-TCTs), distinguished by a carbonyl substitution at the C8 position, represent major contaminants in food and feed worldwide [4]. This group includes deoxynivalenol (DON), nivalenol (NIV), and its prevalent transformation products deoxynivalenol-3-glucoside (DON-3G), 3-acetyl-DON (3-AcDON), and 15-acetyl-DON (15-AcDON). B-TCTs are mainly produced by *Fusarium graminearum* and *F. culmorum*, and they are classified into three chemotypes: NIV), 3-AcDON, and 15-AcDON [5,6,7,8]. Chronic exposure to B-TCTs can induce neurotoxicity, gastrointestinal disorders, and immunotoxicity, posing a substantial risk to human health. B-TCTs contamination is globally prevalent in wheat [9,10,11,12,13]. According to FAO/WHO reports, wheat constitutes the principal dietary source of B-TCTs in most regions, contributing 56–100% of total intake [9]. Therefore, comprehensive risk assessments of B-TCTs in wheat are urgently required.

Several countries have reported exposure assessments of B-TCTs in wheat. The European Food Safety Authority (EFSA) conducted a comprehensive dietary exposure assessment for the total DON group (DON, 3-AcDON, 15-AcDON, and DON-3G) [4]. Comparable assessments of DON and DON-3G exposure from cereals and cereal products have been undertaken in Argentina and the Netherlands [14,15], and exposure to these mycotoxins in grains—including wheat flour—has been evaluated in Egypt [12]. These studies collectively demonstrate that transformation product DON-3G makes a significant contribution to total DON group exposure. Therefore, more attention needs to be paid to its associated health risks [4,12,16]. However, risk assessments involving DON-3G have been conducted only in limited regions in China [17,18]. Hence, it is essential to incorporate DON-3G into total DON group exposure assessments. In addition, DON and NIV frequently co-occur in wheat and can exert combined toxic effects, yet studies investigating their combined exposure remain limited. Given that China is the world’s largest wheat producer, with wheat contributing approximately 86% to human B-TCTs exposure [19,20], a systematic evaluation of both contamination and co-contamination are clearly imperative.

Traditional spatial contamination characteristic analysis and risk assessment are typically based on administrative boundaries [19]. However, such boundaries disrupt natural climatic zones, resulting in inconsistencies in meteorological conditions, wheat cultivation, and dietary patterns. Thus, a more scientifically grounded regional framework is required to accurately characterize B-TCTs contamination. A watershed, as a natural hydrological unit defined by river systems, integrates the water circulation (precipitation, runoff, infiltration, evaporation) and associated material transport processes (e.g., sediment and nutrients) [21,22]. Watershed-based analysis captures the interactions among hydrological, soil, ecological, and anthropogenic processes without the artificial fragmentation of administrative borders, providing a framework that is more consistent with natural and ecological principles.

In this study, we conducted a comprehensive analysis of B-TCTs in 1337 wheat samples collected from China’s primary production regions between 2022 and 2024, with a focus on their watershed-specific distribution, temporal-spatial variation, associated health risks, and key climatic drivers. We systematically investigated the contamination characteristics, performed integrated risk assessments that account for co-exposure and masked metabolites, and identified the dominant climatic drivers—specifically during the flowering-to-harvest period—that influence B-TCTs contamination levels. Against a backdrop in which global climate change may exacerbate mycotoxin contamination, this work provides, for the first time, a watershed-scale overview of the concentrations and exposure risks of B-TCTs and their transformation products in Chinese wheat, thereby forming a comprehensive scientific foundation for the setting of maximum levels, future early warning systems, and targeted mitigation strategies.

## 2. Results and Discussion

### 2.1. Assessing Zoning Methods of Watershed and Administration Division

**Figure 1:**
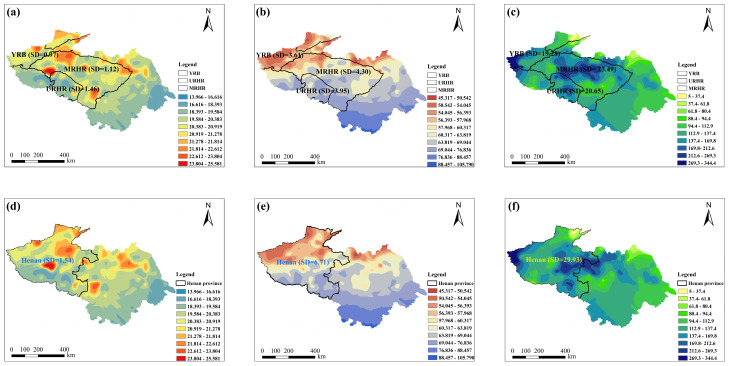
Administration division and watershed-scale division of average temperature (AT), relative humidity (RH), and total precipitation (TP) in Henan province in 2022. (**a**) AT (°C) based on watershed-scale division; (**b**) RH (%) based on watershed-scale division; (**c**) TP (mm) based on watershed-scale division; (**d**) AT (°C) based on administration division; (**e**) RH (%) based on administration division; (**f**) TP (mm) based on administration division. SD means standard deviation.

Given that administrative boundaries segment natural climatic zones, traditional spatial contamination and risk assessments can yield inconsistent data due to variations in meteorology, farming systems, and food consumption. A watershed-based paradigm offers a solution by modeling the complex integrations of hydrological, pedological, ecological, and socioeconomic processes without such artificial divisions, consequently providing a framework that is more consistent with the principles of natural systems. To assess the influence of zoning methodology on climatic homogeneity, standard deviations (SD) of four climatic parameters were calculated under both watershed-based and administrative division frameworks, as illustrated in Figure 1. Under the provincial demarcation in 2022, Henan Province—characterized by a large geographical extent—exhibited substantial intra-provincial climatic variability. The standard deviations of average temperature, relative humidity, and total precipitation were 1.54, 6.71, and 29.93 in 2022 (Figure 1a–c), respectively, indicating low climatic uniformity within the province. By comparison, under the watershed-based framework, the standard deviations of average temperature in the URHR and MRHR (covering parts of Henan Province, including Luoyang and Xinyang) were markedly lower, at 1.46 and 1.12, respectively. Similarly, the standard deviations for relative humidity (3.95 and 4.30) and total precipitation (20.65 and 23.49) were also lower than those derived from provincial delineation (Henan province) (Figure 1d–f). Although the basins extend across multiple administrative regions (unit, provinces), they exhibited strong consistency in key climatic parameters. Therefore, the watershed unit is more appropriate than the administrative unit for analyzing contamination characteristics, as it minimizes the artificial segmentation of natural climatic patterns and provides a more scientific framework for pollutant evaluation.

### 2.2. Spatial and Temporal Distribution Characteristics

#### 2.2.1. Concentration Level and Spatiotemporal Distribution of B-TCTs

**Figure 2:**
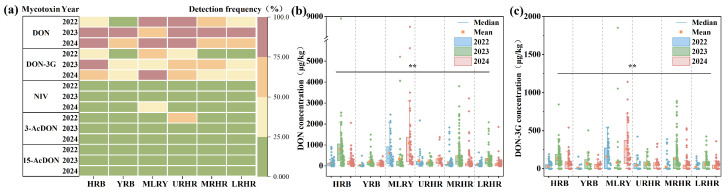
Detection frequency and concentration of B-TCTs in wheat in six river basins in 2022–2024. (**a**) Detection frequency of B-TCTs in wheat in six river basins in 2022–2024, (**b**) concentration of DON in wheat from six river basins in 2022–2024, and (**c**) concentration of DON-3G in wheat from six river basins in 2022–2024. * indicates correlation significance (*p*). Black lines and ** (*p* < 0.01) indicate that the detected mycotoxin levels are statistically significant across all watersheds and three years.

This study investigated the spatiotemporal contamination characteristics of B-TCTs in 1337 wheat samples collected from six major river basins (HRB, YRB, MLRY, URHR, MRHR, and LRHR) between 2022 and 2024. Mean concentrations of DON across the six basins ranged from 22.55 to 1085.61 μg/kg, with detection frequencies between 11.11% and 100.00% (Figure 2a,b). Statistical analysis revealed significant variation in DON concentrations across harvest years (*p* < 0.01) and river basins (*p* < 0.01) (Figure 2b). From 2022 to 2024, mean concentrations of DON in the YRB and LRHR were below 300 μg/kg, indicating relatively low contamination levels. DON concentrations in the MRHR ranged from 119.33 to 441.65 μg/kg. In the HRB, DON contamination levels varied from 107.45 to 778.83 μg/kg, with a highest mean of 778.83 μg/kg observed in 2023. In the URHR, the concentrations of DON varied from 165.97 to 313.52 μg/kg. Notably, the MLRY showed the highest DON contamination, ranging from 317.62 to 1085.61 μg/kg, with mean levels reaching 1085.61 μg/kg in 2024—exceeding the Chinese regulatory limit (1000 μg/kg)—and a detection frequency of 100%.

Elevated DON concentrations may further promote the formation of transformed metabolites. DON-3G, a glucosylated metabolite of DON [23], was detected across all study years and basins at frequencies of 8.33–86.00%, mirroring DON’s spatial and temporal patterns. Mean concentrations of DON-3G ranged from 7.43 to 256.80 μg/kg (Figure 2c). From 2022 to 2024, mean DON-3G concentrations in wheat from the YRB, URHR, and LRHR were all below 100 μg/kg. In the HRB and MRHR, DON-3G contamination levels ranged from 27.43 to 134.91 μg/kg and 18.35–131.24 μg/kg, respectively. Relatively high DON-3G contamination (80.75–256.80 μg/kg) was observed in the YRB, with highest mean contamination levels of 256.80 μg/kg recorded in 2024. The formation of DON-3G is associated with varietal differences in wheat glucosylation capacity [23,24]. Elevated DON concentrations can activate glucosyltransferase enzymes in wheat [25], explaining the frequent co-occurrence of DON-3G with DON hotspots. However, 3-AcDON (detection frequency: 0.00–54.84%; mean concentration: 0.00–26.69 μg/kg) and 15-AcDON (detection frequency: 0.00–24.00%; mean concentration: 0.00–17.55 μg/kg) exhibited lower contamination levels (Figure S1 and Figure 2a).

NIV and DON are both mycotoxins produced by Fusarium species, but they exhibit different distribution characteristics. While DON was widespread, NIV showed a distinct geographic clustering, being primarily detected in the MLRY and Huaihe River Basin (URHR, MRHR, and LRHR), but absent in the HRB and YRB (Figure S1). This pattern suggests that NIV-producing Fusarium species may be confined to the MLRY and Huaihe River Basin. Specifically, NIV-producing *F. asiaticum* strains appear spatially restricted, consistent with prior isolations from Jiangsu, Anhui, Shandong, Hubei, and Henan—but not Hebei provinces [7]. Although overall NIV detection was low, a notable outbreak occurred in the MLRY in 2024 (detection frequency: 44%, mean concentration:119.43 μg/kg) (Figure S1 and Figure 2a).

#### 2.2.2. Co-Occurrence of B-TCTs

**Figure 3:**
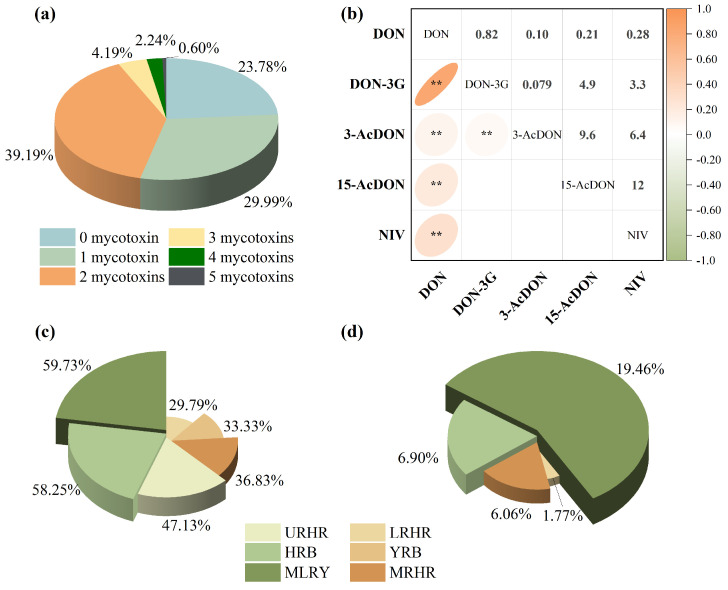
Co-occurrence of B-TCTs contamination in wheat: (**a**) combinations of 1, 2, 3, 4, and 5 contaminants with detection frequencies, (**b**) Spearman correlation of B-TCTs, (**c**) co-occurrence of DON and DON-3G combination, and (**d**) co-occurrence of DON and NIV combination. * indicates correlation significance (*p*). ** means *p* < 0.01.

Co-contamination of B-TCTs was widespread, reflecting the diverse metabolic capabilities of Fusarium species. In the current study, it was observed that 46.22% of the samples were contaminated with two or more mycotoxins, and 7.03% contained three or more (Figure 3a). Spearman correlation analysis revealed a strong positive correlation between DON and DON-3G (rs = 0.82, *p* < 0.01) and a moderate positive correlation between DON and NIV (rs = 0.28, *p* < 0.01) (Figure 3b), indicating distinct co-contamination patterns between these mycotoxin pairs in Chinese wheat. However, the co-contamination rates of DON with DON-3G and DON with NIV exhibited pronounced inter-basin variability (Figure 3c,d). Specifically, DON and DON-3G co-contamination rates were 58.25% and 33.33% in the HRB and YRB, respectively. Co-contamination of DON and NIV was absent in these two basins due to the non-detection of NIV. In contrast, the MLRY exhibited higher co-contamination rates for DON and DON-3G combination (59.73%) and DON and NIV combination (19.46%). Lower yet notable co-contamination levels were observed in the Huaihe River sub-basins, where the URHR, MRHR, and LRHR showed DON and DON-3G rates of 47.13%, 36.83%, and 29.79%, and DON and NIV rates of 6.90%, 6.06%, and 1.77%, respectively.

B-TCTs contamination in wheat showed significant spatial heterogeneity across the river basins. The MLRY showed the highest co-contamination frequencies, primarily involving DON, DON-3G, and NIV, followed by the URHR. In contrast, MRHR, HRB, and YRB were predominantly contaminated with DON alone at relatively lower levels. These distinct spatial patterns in contamination levels and detection frequencies underscore the influence of regional climatic factors, emphasizing the need for spatially targeted risk assessments.

### 2.3. Human Health Risk Assessment

#### 2.3.1. Health Risk Assessment to Total DON Group

Chronic exposure to total DON group (tDON include DON, DON-3G, 3-AcDON, and 15-AcDON) poses potential health risks to humans. In this study, chronic dietary exposure to tDON and NIV was estimated for different age groups using a deterministic assessment approach (Tables S1 and S2). In 2022, the mean and median *HQ* values for tDON among children aged 3–6 years in the MLRY and URHR ranged from 1.06 to 1.50, exceeding the safety threshold value (*HQ* = 1) and indicating a potential health risk. In contrast, other age groups within these basins and all groups across other regions demonstrated acceptable levels of tDON exposure. In 2023, all age groups in the HRB exhibited elevated chronic exposure risks, with mean *HQ* values ranging from 1.48 to 2.95. Additionally, in the MRHR, children (3–6 years), schoolers (7–12 years), and adults (18–59 years) faced notable chronic exposure risks, with the mean *HQ* values between 1.08 and 2.13. However, adolescents (13–17 years) and older adults (≥60 years) in the same basin exhibited lower risks due to their comparatively lower wheat consumption. In the LRHR, only children aged 3–6 years exhibited a marginally elevated risk (mean *HQ* = 1.01), whereas other age groups had lower exposure levels, indicating no appreciable health risks from wheat consumption. In 2024, the mean *HQ* values ranged from 0.99 to 2.66 across all age groups, with children aged 3–6 years in the MLRY showing the highest tDON exposure risk (*HQ* = 2.66). Furthermore, mean and median *HQ* values (1.30–1.33) for children aged 3–6 years in the URHR exceeded limited values, suggesting that tDON exposure levels were unacceptable. Based on the above analysis, children aged 3–6 years consistently exhibited the highest susceptibility to the adverse health effects caused by tDON across all river basins and study years. This can be attributed to their highest wheat intake per unit body weight [26]. Similar exposure patterns have been reported in other countries [27,28,29]. Children aged 3–6 years should remain the primary focus of risk assessments, and stricter tDON limits in children’s foods are recommended.

**Figure 4:**
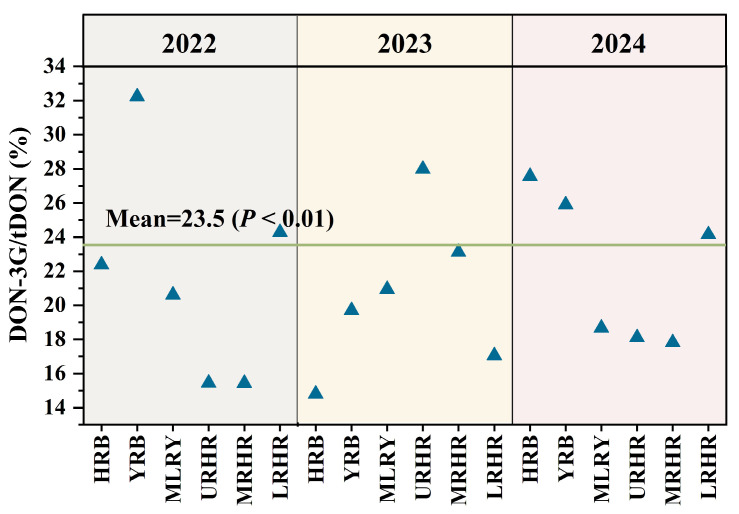
Contribution of DON-3G to tDON exposure risk in six river basins in 2022–2024.

Current DON exposure assessments primarily consider DON and its acetylated derivatives (3-AcDON and 15-AcDON), often neglecting the significant contribution of DON-3G to tDON exposure. Notably, our result show that the contributions of DON-3G to tDON exposure varied across all study years and basins (Figure 4). DON-3G contributed approximately 14.80–32.23% to tDON exposure, with an average of 23.5% (*p* < 0.01) in China, closely aligning with the EFSA estimate of 20% [4]. The masked glucoside metabolite DON-3G significantly increased tDON exposure.

#### 2.3.2. Health Risk Assessment to Total DON Group and NIV

DON and NIV are both B-TCTs with closely related chemical structures, differing only by the substituent at the C4 position, which results in comparable toxicological profiles [30,31]. Several studies have demonstrated that DON and NIV exert combined toxic effects in both in vitro and in vivo models [32,33,34,35]. Both DON and NIV are produced by Fusarium species and frequently co-contaminate wheat in specific areas (e.g., MLRY). Although the exposure risk from individual mycotoxins may be limited, their simultaneous presence raises significant toxicological concern. Co-occurrence of multiple mycotoxins can substantially elevate cumulative exposure risk, potentially exceeding safety thresholds and posing a serious threat to human health.

The combined exposure risk, expressed as the *HI* for tDON and NIV groups, is presented in Table 1. In 2022, the mean *HI* values for children aged 3–6 years in both the MLRY and URHR exceeded 1, indicating potential health risks, whereas combined exposure levels in other basins and age groups remained relatively low. In 2023, all age groups in the MRHR—except adolescents (13–17 years) and older adults (≥60 years)—exhibited *HI* values ranging from 1.08 to 2.14, indicating elevated combined exposure. Additionally, children aged 3–6 years in the LRHR exhibited a notable combined exposure risk (*HI* = 1.02). In 2024, all age groups in the MLRY exhibited mean *HI* values above 1 (1.06–2.85), reflecting persistent combined exposure risks. Children aged 3–6 years in the URHR were also at risk, with an *HI* value of 1.36. In contrast, mean and median *HI* values in other basins remained below the safety threshold. Notably, the high-consumption population (P95) exhibited elevated *HI* values across most basins, indicating a higher cumulative risk. Although DON was the dominant contributor to the combined exposure risk, the contribution of NIV across the study areas ranged from 0 to 13.12% with the maximum contribution observed in the MRHR in 2024. Despite this wide range, NIV’s role was generally minor overall; however, its notable impact in specific regions such as the MRHR underscores the need for focused monitoring and risk management.

### 2.4. Correlation Among Climatic Factors and B-TCTs in Wheat

**Figure 5:**
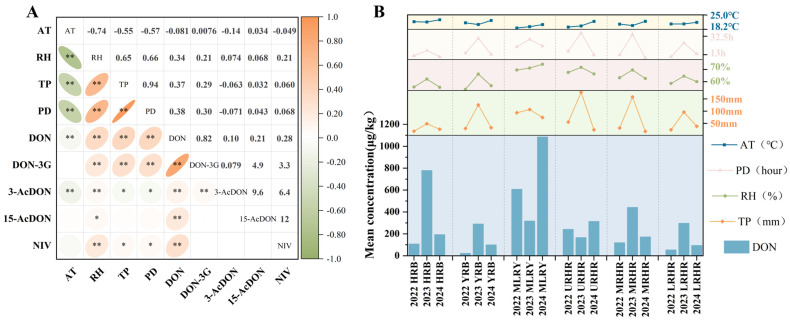
(**a**) Spearman correlation analysis of B-TCTs in wheat with climatic factors; (**b**) Mean values of climatic factors and mean concentration of DON in wheat from six river basins in 2022–2024. * indicates correlation significance (*p*). * means *p* < 0.05, ** means *p* < 0.01.

B-TCTs contamination levels and risk assessment mentioned above vary significantly across different years and river basins. To further investigate the key climatic factors influencing B-TCTs contamination, this study analyzed the correlations between contamination levels in Chinese wheat and four climatic factors during the flowering-to-harvest period: average temperature (AT), relative humidity (RH), total precipitation (TP), and precipitation duration (PD) (Figure 5a). The results revealed that DON concentrations in Chinese wheat were significantly and positively correlated with RH (rs = 0.34, *p* < 0.01), TP (rs = 0.37, *p* < 0.01), and PD (rs = 0.38, *p* < 0.01), whereas their correlation with AT was weak (rs < 0.1, *p* < 0.01). This weak temperature dependence may be attributed to the low temperature variability in the study area (coefficients of variation = 0.06), suggesting that minor thermal fluctuations were insufficient to drive significant changes in mycotoxin accumulation. The contamination level of DON-3G was highly dependent on its parent mycotoxin—DON. Moreover, in basins where DON concentrations exhibited significant correlations with climatic variables, DON-3G also showed parallel associations (Figure 5a). Other B-TCTs were either undetected or detected at relatively low frequencies, which precluded a robust assessment of their correlations with meteorological factors.

Further analysis of basin-specific patterns (Figure 5b) demonstrated that during 2022–2024, years with higher DON contamination levels in the YRB, MRHR, and LRHR, were characterized by elevated RH, TP, and PD. For instance, in the MRHR, these three climatic variables reached their highest values in 2023, coinciding with the highest DON level (441.65 μg/kg). Similarly, in the HRB, the flowering-to-harvest period of 2023 exhibited substantially higher RH (62.28%), TP (48.33 mm), and PD (17 h) than in 2022 and 2024, which coincided with the highest DON concentrations (778.83 μg/kg). Interestingly, a distinct pattern was observed in the MLRY, where the lowest TP in 2024 coincided with the highest values of AT and RH, corresponding to the highest contamination level of DON. This pattern suggests that the synergistic effects of high temperature and humidity amplified DON biosynthesis. This interpretation aligns with previous studies reporting that temperature fluctuations play a crucial regulatory role in mycotoxin production under high-humidity condition [36].

Overall, RH, TP, and PD exerted significant positive effects on DON accumulation across most years and basins, whereas temperature exhibited a relatively limited impact due to its small variability within the study region. These findings indicate that multiple meteorological factors collectively regulate mycotoxin biosynthesis and accumulation, leading to spatially heterogeneous contamination patterns across basins. Future studies should integrate microbiological and agrometeorological approaches to elucidate the multi-scale mechanisms underlying climate–toxin interactions and to develop region-specific prediction and control strategies for more precise mycotoxin risk management.

## 3. Conclusions

This study provides the first systematic, watershed-scale analysis of B-TCTs and their transformation products in wheat over a three-year period (2022–2024). By combining B-TCTs contamination data with wheat consumption patterns across major Chinese river basins, we comprehensively assessed the spatial-temporal characteristics and dietary exposure risks of B-TCTs. Our results show that watershed-based zoning better captures the relationship between mycotoxin contamination and climatic drivers. DON and its transformed metabolite DON-3G were the predominant contaminants, with DON-3G making a significant contribution to total DON group exposure. NIV contamination showed distinct regional patterns and presented a co-contamination risk with DON. Exposure assessment revealed potential health risks in specific basins—such as the MLRY and HRB—during certain years, especially for children aged 3–6 years and high-consumption groups. Among meteorological factors, RH, TP and PD were key drivers of DON contamination. It is noteworthy that the key driving roles of humidity and precipitation identified in this study suggest that under the context of global climate change, characterized by increasing frequency of extreme weather events and alterations in precipitation patterns during growing seasons, the risk landscape of B-TCTs contamination in China’s major wheat-producing regions may further evolve. We recommend that future mycotoxin regulations include DON-3G in risk assessments and implement targeted controls in high-risk basins (e.g., MLRY, HRB) and for vulnerable groups (children). Based on our findings that high relative humidity and prolonged precipitation during the post-flowering period are key drivers of contamination, we suggest developing dynamic early warning systems that integrate weather forecasts for this critical window. Such systems would enable precise agriculture interventions, such as the timely application of biocontrol agents or optimized fungicide schedules, specifically for high-risk areas. Furthermore, breeding programs in these basins could prioritize wheat varieties with enhanced resistance to Fusarium infection or a lower capacity for DON-3G formation. 

## 4. Materials and Methods

### 4.1. Study Area and Sample Collection

**Figure 6:**
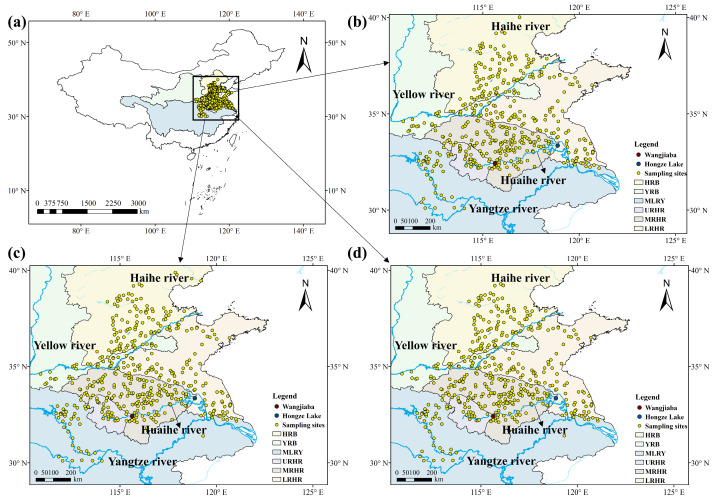
Location and distribution of wheat sampling sites for the study area. (**a**) Map showing the sampling sites of wheat across China in 2022–2024, (**b**) wheat sampling sites in 2022, (**c**) wheat sampling sites in 2023, and (**d**) wheat sampling sites in 2024.

The study areas were selected based on an integrated assessment of river basin boundaries, dominant wheat cultivation systems, and anthropogenic influences (http://www.mwr.gov.cn/szs/mcjs, accessed on 19 January, 2026). Four primary river basins were identified: the Haihe, Yellow, Yangtze, and Huaihe River Basins. The geographical boundaries of these basins were obtained from the respective water authority websites (http://www.hwcc.gov.cn/; http://www.yrcc.gov.cn/; http://www.cjw.gov.cn/; http://www.hrc.gov.cn/, accessed on 19 January, 2026). For detailed spatial analysis, the study area was subdivided into six regions: the Haihe River Basin (HRB), Yellow River Basin (YRB), Middle and Lower Reaches of the Yangtze River Basin (MLRY), Upper Reaches of the Huaihe River (URHR), Middle Reaches of the Huaihe River (MRHR), and Lower Reaches of the Huaihe River (LRHR), encompassing the Yishusi River Basin and the Shandong Peninsula (Figure 6, Text S1).

A total of 1337 wheat samples were collected from different river basins during the monitoring period, of which 437, 456, and 444 were in 2022, 2023, and 2024, respectively (Figure 6). All samples were collected at the fully mature stage during May and June. Approximately 1 kg of wheat was collected per sampling point. All collected wheat samples were stored in self-sealing polyethylene bags and sent back to the laboratory for further chemical analysis.

Accurate matching of climatic factors to each sampling location was essential. Monthly climate data from different sites were obtained from local meteorological stations. Three factors for climate information, including temperature, relative humidity, and precipitation from 0:00 to 24:00, were acquired from the official website of the National Meteorological Information Center of China. The missing data were obtained from the official websites of the meteorological offices of each province and these data sets were standardized by the originating department to ensure comparability. Climate data for 2022–2024 were aligned with the geographic coordinates of each wheat sampling site using a Python-based algorithm that assigned data from the nearest meteorological station. Four climatic parameters during the flowering-to-harvest period were analyzed: average temperature (AT), relative humidity (RH), total precipitation (TP), and precipitation duration (PD), as illustrated in Figure S2.

### 4.2. Sample Pretreatment and Analysis

All samples were assigned unique identification codes and sent to the Academy of National Food and Strategic Reserves Administration (No. 11 Baiwanzhuang Str, Xicheng District, Beijing, China) for analysis. In total, 1 kg of each wheat sample was weighed and ground using an electronic grander (TDW-4500, TXTB LAB INSTRUMENT, Beijing, China) and the processed wheat was passed through mesh with diameter of 425 μm. Each sample was ground into powder, homogenized by shaking it thoroughly inside the sealed bag, and stored in sealed polyethylene bags at 4 °C until analysis. Sample preparation followed the protocol described by Wu [37] and Ye [38], with minor modifications. For the extraction, 5 g of each sample was placed in a screw cap centrifugal tube with 20 mL of the extraction solvent: acetonitrile analytical reagent grade 99.99% purchased from Merck (Darmstadt, Germany), bi-distilled water and acetic acid analytical reagent grade 98–100% obtained from Fischer Scientific (Waltham, MA, USA) in 70:29:1 proportion, respectively. Subsequently, the sample was mixed for 20 min with a multi-tube vortex mixer purchased from SCDEALL (Beijing, China). Then, the mixture was centrifuged at 7000 rpm for 5 min in a centrifuge purchased from EPPENDORF (Hamburg, Germany). An aliquot of 0.5 mL supernatant was mixed with 0.5 mL of water, vortexed for 1 min, and centrifuged at 10,000× *g* for 10 min at 4 °C in a centrifuge purchased from EPPENDORF (Hamburg, Germany). The supernatant was filtered by a 0.22 μm PTFE filters purchased from PALL (New York, NY, USA). A 180 μL aliquot of the filtrate was combined with 20 μL of isotopically labeled internal standard Purchased from IniKem BioPharmaTech Co., Ltd. (Qingdao, China), mixed thoroughly, and injected into the LC-MS/MS system purchased from Fischer Scientific (Waltham, MA, USA) for quantification. 

### 4.3. Risk Assessment

A deterministic model was employed to quantitatively assess the dietary exposure risk of B-TCTs through wheat consumption. Concentration data for B-TCTs in wheat were obtained from the Academy of National Food and Strategic Reserves Administration. Measured concentrations were directly used in the analysis. The LOD values for B-TCTs are shown in Table S3. For samples with concentrations below the limit of detection (LOD), the substitution method recommended by EFSA was adopted: if fewer than 60% of values were below the LOD, they were replaced with ½ LOD; if more than 60% were below the LOD, values were recognized as 0 [39]. Previous studies have shown that preprocessing steps such as washing, sorting, dehulling, and milling significantly reduce mycotoxin concentrations in wheat [40,41]. The processing factor (PF), which quantifies concentration changes during wheat processing, is defined as the ratio between the concentration in the processed product and in raw wheat [42]. PF is a critical parameter for characterizing dietary exposure and chemical residues in processed foods [43,44] and was applied in this study to refine exposure assessment.

Wheat consumption data were derived from the 2019 China National Nutrition and Health Survey, which included 45,192 participants across 20 provinces. Wheat consumption was stratified by river basin and age group to estimate toxin exposure risks. Body weight data were also incorporated to support dietary risk assessment. Dietary exposure to individual mycotoxin was estimated using the following equation:

(1) $$EDI=\left(C\times F\times PF\right)/BW$$

where *EDI* is the estimated daily intake (μg/kg bw/d); *C* represents the concentration of specific mycotoxins in wheat (μg/kg), with DON defined as the sum of DON, DON-3G, 3-Ac-DON, and 15-Ac-DON; *F* denotes wheat consumption (kg/d) expressed as mean, median (P50), and 95th percentile (P95). The details are shown in Table S4. *PF* is the wheat processing factor (0.35) as reported by [44] and *BW* is the average body weight (kg) of different age groups.

To characterize risk, the estimated dietary exposures were compared with toxicological reference values [45]. The tolerable daily intake (TDI, μg/kg bw/d) is defined as the maximum daily toxin intake without adverse health effects in humans. The Joint FAO/WHO Expert Committee on Food Additives (JECFA) and EFSA have established a provisional maximum tolerable daily intake (PMTDI/TDI) of 1 μg/kg bw/d for DON, 3-AcDON, 15-AcDON, and DON-3G, and 1.2 μg/kg bw/d for NIV [4,46].

The hazard quotient (*HQ*) was used to assess the health risk of each B-TCT and was calculated as [47]:

*HQ* = *EDI*/TDI (2)

To evaluate the potential combined effects of B-TCTs, the individual *HQ* for each mycotoxin were aggregated and presented as a Hazard Index (*HI*), calculated as follows:

(3) $$\mathrm{HI}=\sum_{1}^{n}{\mathrm{HQ}}_{i}$$

where *HI* < 1 indicates negligible risk, while *HI* > 1 suggest a potential adverse health risk [45].

### 4.4. Statistical Analysis

All statistical analyses were performed using ArcGIS 10.8.2, Origin 2022, and Python 3.7. The Kruskal–Wallis H test and Dunn’s post hoc test were employed for non-parametric comparisons. Statistical significance was set at *p* < 0.05 and *p* < 0.01.

## Figures and Tables

**Table 1:** Hazard index (*HI*) of tDON and NIV in wheat from six river basins in 2022–2024.

River Basin	Age Group	Hazard Index (Contribution of NIV)								
		2022			2023			2024		
		Mean	Median	P95	Mean	Median	P95	Mean	Median	P95
HRB	3–6 years	0.46 (0%)	0.41 (0%)	0.98 (0%)	2.95 (0%)	2.66 (0%)	6.28 (0%)	0.86 (0%)	0.78 (0%)	1.83 (0%)
	7–12 years	0.33 (0%)	0.32 (0%)	0.58 (0%)	2.09 (0%)	2.04 (0%)	3.72 (0%)	0.61 (0%)	0.59 (0%)	1.08 (0%)
	13–17 years	0.24 (0%)	0.22 (0%)	0.48 (0%)	1.53 (0%)	1.43 (0%)	3.07 (0%)	0.45 (0%)	0.42 (0%)	0.89 (0%)
	18–59 years	0.23 (0%)	0.22 (0%)	0.44 (0%)	1.48 (0%)	1.40 (0%)	2.80 (0%)	0.43 (0%)	0.41 (0%)	0.82 (0%)
	≥60 years	0.25 (0%)	0.24 (0%)	0.48 (0%)	1.61 (0%)	1.52 (0%)	3.05 (0%)	0.47 (0%)	0.44 (0%)	0.89 (0%)
	Total population	0.24 (0%)	0.23 (0%)	0.47 (0%)	1.56 (0%)	1.48 (0%)	3.03 (0%)	0.46 (0%)	0.43 (0%)	0.88 (0%)
YRB	3–6 years	0.09 (0%)	0.09 (0%)	0.21 (0%)	0.81 (0%)	0.78 (0%)	1.81 (0%)	0.33 (0%)	0.31 (0%)	0.73 (0%)
	7–12 years	0.06 (0%)	0.06 (0%)	0.13 (0%)	0.56 (0%)	0.52 (0%)	1.13 (0%)	0.23 (0%)	0.21 (0%)	0.46 (0%)
	13–17 years	0.05 (0%)	0.05 (0%)	0.11 (0%)	0.45 (0%)	0.42 (0%)	0.92 (0%)	0.18 (0%)	0.17 (0%)	0.37 (0%)
	18–59 years	0.04 (0%)	0.04 (0%)	0.10 (0%)	0.39 (0%)	0.33 (0%)	0.89 (0%)	0.16 (0%)	0.13 (0%)	0.36 (0%)
	≥60 years	0.05 (0%)	0.04 (0%)	0.11 (0%)	0.40 (0%)	0.35 (0%)	0.92 (0%)	0.16 (0%)	0.14 (0%)	0.37 (0%)
	Total population	0.05 (0%)	0.04 (0%)	0.11 (0%)	0.41 (0%)	0.35 (0%)	0.97 (0%)	0.17 (0%)	0.14 (0%)	0.39 (0%)
MLRY	3–6 years	1.54 (2.75%)	1.54 (2.76%)	3.29 (2.76%)	0.82 (3.34%)	0.82 (3.35%)	1.74 (3.35%)	2.85 (6.72%)	2.85 (6.73%)	6.08 (6.73%)
	7–12 years	0.95 (2.75%)	0.89 (2.74%)	2.16 (2.75%)	0.5 (3.36%)	0.48 (3.31%)	1.14 (3.35%)	1.74 (6.74%)	1.64 (6.74%)	3.98 (6.73%)
	13–17 years	0.63 (2.75%)	0.60 (2.77%)	1.55 (2.75%)	0.33 (3.35%)	0.32 (3.31%)	0.82 (3.36%)	1.16 (6.73%)	1.10 (6.76%)	2.86 (6.73%)
	18–59 years	0.73 (2.75%)	0.61 (2.76%)	1.77 (2.75%)	0.38 (3.37%)	0.32 (3.36%)	0.93 (3.36%)	1.35 (6.71%)	1.13 (6.72%)	3.27 (6.72%)
	≥60 years	0.58 (2.74%)	0.49 (2.75%)	1.62 (2.75%)	0.3 (3.37%)	0.26 (3.37%)	0.86 (3.35%)	1.06 (6.70%)	0.91 (6.71%)	3.00 (6.72%)
	Total population	0.67 (2.74%)	0.56 (2.78%)	1.78 (2.75%)	0.35 (3.34%)	0.30 (3.30%)	0.94 (3.34%)	1.22 (6.74%)	1.04 (6.69%)	3.28 (6.73%)
URHR	3–6 years	1.11 (4.89%)	1.14 (4.89%)	1.75 (4.90%)	0.77 (0%)	0.79 (0%)	1.21 (0%)	1.36 (4.67%)	1.39 (4.65%)	2.14 (4.67%)
	7–12 years	0.75 (4.93%)	0.77 (4.92%)	1.56 (4.91%)	0.52 (0%)	0.54 (0%)	1.08 (0%)	0.92 (4.66%)	0.94 (4.67%)	1.92 (4.65%)
	13–17 years	0.62 (4.92%)	0.60 (4.92%)	0.99 (4.90%)	0.43 (0%)	0.42 (0%)	0.69 (0%)	0.77 (4.66%)	0.73 (4.69%)	1.22 (4.65%)
	18–59 years	0.58 (4.88%)	0.54 (4.92%)	1.06 (4.91%)	0.40 (0%)	0.37 (0%)	0.74 (0%)	0.70 (4.69%)	0.66 (4.66%)	1.31 (4.65%)
	≥60 years	0.53 (4.93%)	0.53 (4.90%)	0.97 (4.88%)	0.37 (0%)	0.37 (0%)	0.67 (0%)	0.65 (4.66%)	0.65 (4.63%)	1.19 (4.66%)
	Total population	0.59 (4.89%)	0.55 (4.94%)	1.11 (4.92%)	0.41 (0%)	0.38 (0%)	0.78 (0%)	0.72 (4.65%)	0.68 (4.63%)	1.37 (4.66%)
MRHR	3–6 years	0.63 (7.79%)	0.60 (7.8%)	1.25 (7.81%)	2.14 (0.45%)	2.04 (0.45%)	4.26 (0.45%)	0.92 (13.02%)	0.87 (13.02%)	1.84 (12.95%)
	7–12 years	0.37 (7.76%)	0.38 (7.76%)	0.72 (7.87%)	1.26 (0.45%)	1.29 (0.45%)	2.47 (0.45%)	0.54 (12.95%)	0.55 (13.04%)	1.06 (13.02%)
	13–17 years	0.27 (7.75%)	0.26 (7.95%)	0.51 (7.90%)	0.92 (0.44%)	0.90 (0.45%)	1.77 (0.45%)	0.39 (13.12%)	0.39 (12.97%)	0.76 (12.98%)
	18–59 years	0.31 (7.86%)	0.33 (7.75%)	0.57 (7.77%)	1.08 (0.45%)	1.10 (0.45%)	1.96 (0.45%)	0.47 (12.85%)	0.47 (13.06%)	0.84 (13.01%)
	≥60 years	0.29 (7.76%)	0.29 (7.91%)	0.56 (7.87%)	0.99 (0.45%)	1.01 (0.45%)	1.95 (0.45%)	0.43 (13.05%)	0.44 (12.98%)	0.84 (12.95%)
	Total population	0.31 (7.75%)	0.31 (7.76%)	0.60 (7.85%)	1.06 (0.45%)	1.06 (0.45%)	2.05 (0.45%)	0.46 (12.95%)	0.46 (12.97%)	0.88 (12.95%)
LRHR	3–6 years	0.25 (0%)	0.23 (0%)	0.55 (0%)	1.02 (1.02%)	0.93 (1.03%)	2.27 (1.02%)	0.39 (2.52%)	0.36 (2.50%)	0.87 (2.51%)
	7–12 years	0.18 (0%)	0.15 (0%)	0.40 (0%)	0.72 (1.03%)	0.64 (1.03%)	1.63 (1.03%)	0.28 (2.51%)	0.25 (2.50%)	0.63 (2.51%)
	13–17 years	0.14 (0%)	0.13 (0%)	0.31 (0%)	0.58 (1.02%)	0.53 (1.03%)	1.27 (1.02%)	0.22 (2.56%)	0.21 (2.48%)	0.48 (2.54%)
	18–59 years	0.13 (0%)	0.11 (0%)	0.30 (0%)	0.53 (1.02%)	0.45 (1.04%)	1.24 (1.02%)	0.20 (2.57%)	0.17 (2.54%)	0.47 (2.53%)
	≥60 years	0.13 (0%)	0.12 (0%)	0.29 (0%)	0.52 (1.03%)	0.48 (1.02%)	1.17 (1.03%)	0.19 (2.55%)	0.18 (2.52%)	0.45 (2.51%)
	Total population	0.13 (0%)	0.11 (0%)	0.32 (0%)	0.54 (1.03%)	0.46 (1.02%)	1.29 (1.03%)	0.21 (2.53%)	0.17 (2.57%)	0.50 (2.49%)

## Data Availability

The original contributions presented in the study are included in the article/Supplementary Materials, further inquiries can be directed to the corresponding authors.

## Supplementary Materials

The following supporting information can be downloaded at: https://www.mdpi.com/article/10.3390/toxins18030150/s1, Figure S1: Mean concentration of NIV, 3-AcDON, and 15-AcDON in wheat from six river basins in 2022–2024; Figure S2: Spatial distribution of four climate factors of six river basins in 2022–2024; Figure S3: Linear regression analysis between DON and DON-3G concentrations; Table S1: Hazard quotient (mean, median, P95) of DONs of different age group in six river basins in 2022–2024; Table S2: Hazard quotient (mean, median, P95) of NIV of different age groups in six river basins in 2022–2024; Table S3: The LOD and LOQ values for B-TCTs; Table S4: Wheat consumption (mean, median, and P95 in g/day) in the six river basins. Text S1: Explanatory notes on the division of the Huaihe River Basin.
