# Supplementary material for: Occurrence, Distribution Characteristics, Risk Assessment, and Climatic Drivers of Type B Trichothecenes and Their Transformation Products in Major Wheat-Producing Areas of China
Source: Toxins (Basel). 2026 Mar 21;18(3):150. doi: 10.3390/toxins18030150 (PMC13030218; doi:10.3390/toxins18030150)
Supplement: Supplementary file 1 [file toxins-18-00150-s001.zip › toxins-4201027-supplementary.pdf]

Occurrence, Distribution Characteristics, Risk Assessment, and Climatic Drivers of Type B Trichothecenes and Their Transformation Products in Major Wheat-Producing Areas of China

Jie Wang [1,2,†], Yu Wu [2,†], Di Cai [2], Li Li [2], Songshan Wang [2], Yu Zhang [2,3], Xiaomin Han [4,5,*], Songxue Wang [2], Leiqing Pan [1,*] and Jin Ye [2,*]

[1] College of Food Science and Technology, Nanjing Agricultural University, Nanjing 210095, China; 2023808109@stu.njau.edu.cn
[2] NFSRA Key Laboratory of Grain and Oil Quality and Safety, Academy of National Food and Strategic Reserves Administration, Beijing 100037, China; wyu@ags.ac.cn (Y.W.); cd@ags.ac.cn (D.C.); ll@ags.ac.cn (L.L.); wss@ags.ac.cn (S.W.); csuftzy@163.com (Y.Z.); wsx@ags.ac.cn (S.W.)
[3] College of Food Science and Engineering, Central South University of Forestry and Technology, Changsha 410004, China
[4] NHC Key Laboratory of Food Safety Risk Assessment, China National Center for Food Safety Risk Assessment, Beijing 100021, China
[5] School of Public Health, Southern Medical University, Guangzhou 510515, China
* Correspondence: hanxiaomin@cfsa.net.cn (X.H.), pan_leiqing@njau.edu.cn (L.P.), yj@ags.ac.cn (J.Y.)
† These authors contributed equally to this work.

Figure S1. Mean concentration of NIV, 3-AcDON, and 15-AcDON in wheat from six river basins in 2022-2024

Figure S2. Spatial distribution of four climate factors of six river basins in 2022-2024

Figure S3. Linear regression analysis between DON and DON-3G concentrations

Table S1. Hazard quotient (mean, median, P95) of DONs of different age group in six river basins in 2022-2024

Table S2. Hazard quotient(mean, median, P95) of NIV of different age group in six river basins in 2022-2024

Table S3. The LOD and LOQ values for B-TCTs.

Table S4. wheat consumption (mean, median, and P95 in g/day) for each age group in the six river basins.

Text S1 Explanatory notes on the division of the Huaihe River Basin

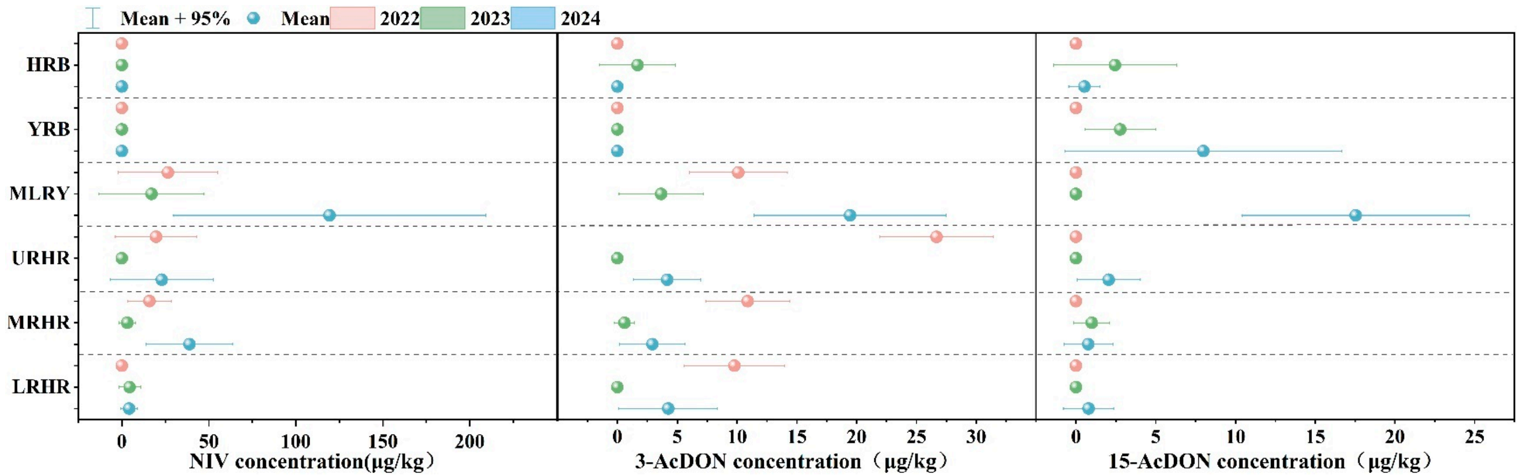

Figure S1. Mean concentration of NIV, 3-AcDON, and 15-AcDON in wheat from six river basins in 2022-2024

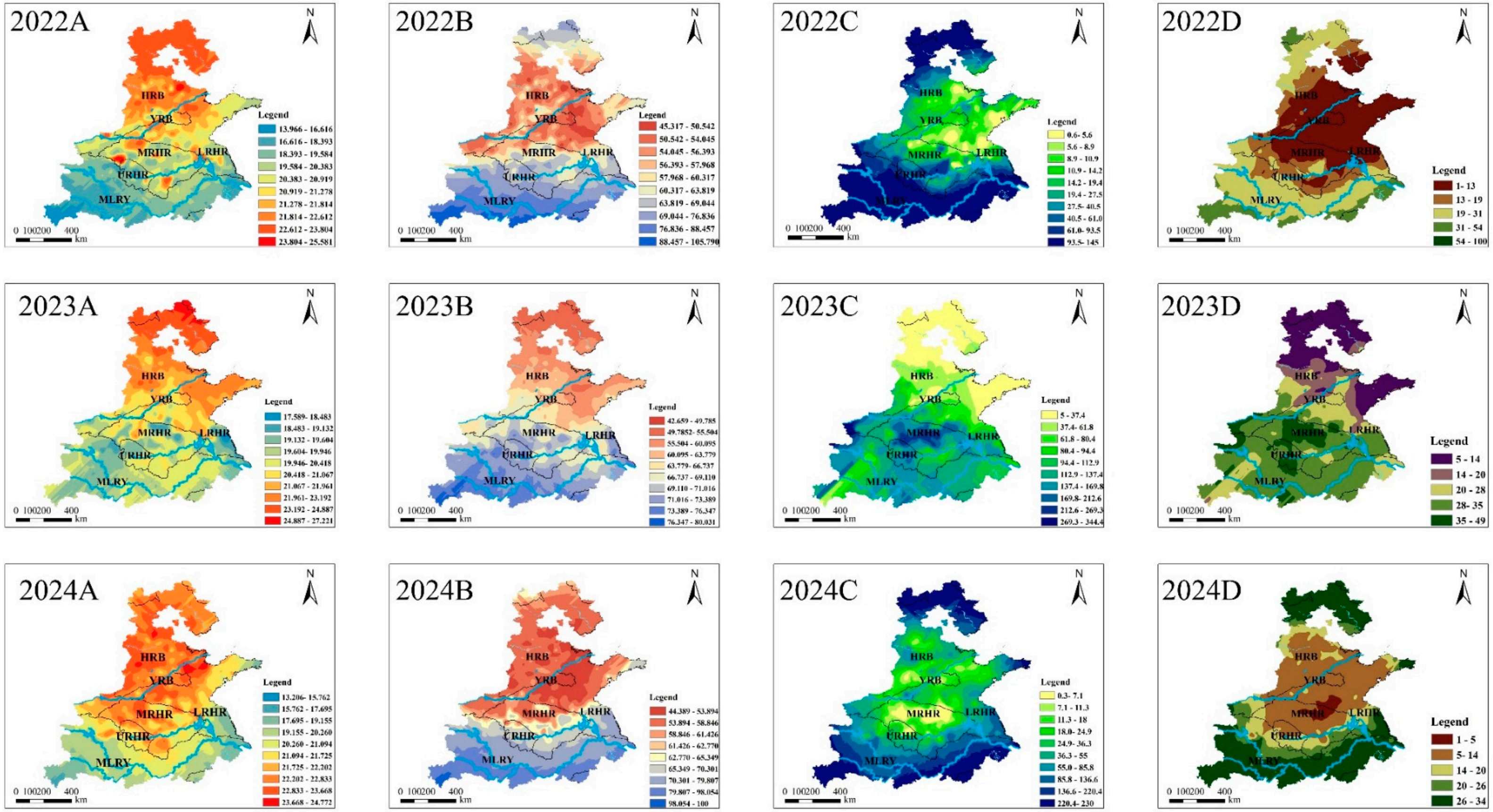

Figure S2. Spatial distribution of four climate factors of six river basins in 2022-2024. A: average temperature(AT) (°C); B: relative humidity (%); C: total precipitation (millimeters, mm); D: precipitation duration (hour).

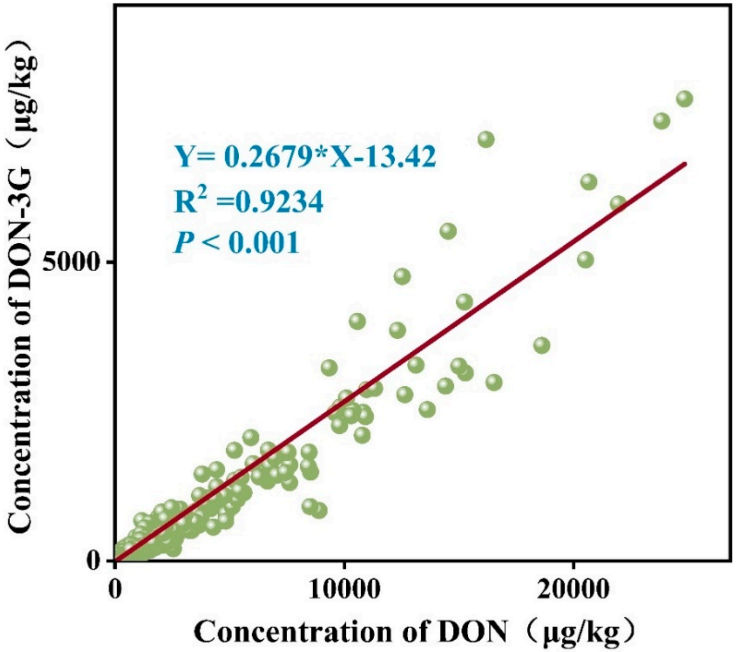

Figure S3. Linear regression analysis between DON and DON-3G concentrations

Table S1. Hazard quotient (mean, median, P95) of DONs of different age group in six river basins in 2022-2024

River basin	Age group	Total DON HQ （DON HQ）								
		2022			2023			2024		
		Mean	Median	P95	Mean	Median	P95	Mean	Median	P95
HRB	3-6years	0.46(0.36)	0.41(0.32)	0.98(0.76)	2.95(2.50)	2.66(2.25)	6.28(5.32)	0.86(0.62)	0.78(0.56)	1.83(1.32)

	7-12years	0.33(0.25)	0.32(0.25)	0.58(0.45)	2.09(1.77)	2.04(1.73)	3.72(3.15)	0.61(0.44)	0.59(0.43)	1.08(0.78)
	13-17years	0.24(0.18)	0.22(0.17)	0.48(0.37)	1.53(1.29)	1.43(1.21)	3.07(2.60)	0.45(0.32)	0.42(0.30)	0.89(0.65)
	18-59years	0.23(0.18)	0.22(0.17)	0.44(0.34)	1.48(1.25)	1.40(1.18)	2.80(2.38)	0.43(0.31)	0.41(0.29)	0.82(0.59)
	⩾60years	0.25(0.19)	0.24(0.18)	0.48(0.37)	1.61(1.36)	1.52(1.29)	3.05(2.58)	0.47(0.34)	0.44(0.32)	0.89(0.64)
	Total population	0.24(0.19)	0.23(0.18)	0.47(0.37)	1.56(1.32)	1.48(1.25)	3.03(2.57)	0.46(0.33)	0.43(0.31)	0.88(0.64)
YRB	3-6years	0.09(0.06)	0.09(0.06)	0.21(0.14)	0.81(0.65)	0.78(0.62)	1.81(1.44)	0.33(0.23)	0.31(0.22)	0.73(0.50)
	7-12years	0.06(0.04)	0.06(0.04)	0.13(0.09)	0.56(0.44)	0.52(0.41)	1.13(0.90)	0.23(0.16)	0.21(0.14)	0.46(0.31)
	13-17years	0.05(0.04)	0.05(0.03)	0.11(0.07)	0.45(0.36)	0.42(0.33)	0.92(0.73)	0.18(0.13)	0.17(0.12)	0.37(0.26)
	18-59years	0.04(0.03)	0.04(0.03)	0.10(0.07)	0.39(0.31)	0.33(0.26)	0.89(0.71)	0.16(0.11)	0.13(0.09)	0.36(0.25)
	⩾60years	0.05(0.03)	0.04(0.03)	0.11(0.07)	0.40(0.32)	0.35(0.28)	0.92(0.73)	0.16(0.11)	0.14(0.10)	0.37(0.26)
	Total population	0.05(0.03)	0.04(0.03)	0.11(0.08)	0.41(0.32)	0.35(0.28)	0.97(0.77)	0.17(0.11)	0.14(0.10)	0.39(0.27)
MLRY	3-6years	1.50(1.17)	1.50(1.17)	3.20(2.50)	0.79(0.62)	0.79(0.62)	1.68(1.32)	2.66(2.09)	2.66(2.09)	5.67(4.46)
	7-12years	0.92(0.72)	0.87(0.68)	2.10(1.64)	0.48(0.38)	0.46(0.36)	1.10(0.86)	1.62(1.28)	1.53(1.21)	3.71(2.92)
	13-17years	0.61(0.48)	0.58(0.46)	1.51(1.18)	0.32(0.25)	0.31(0.24)	0.79(0.62)	1.08(0.85)	1.03(0.81)	2.67(2.10)
	18-59years	0.71(0.55)	0.59(0.46)	1.72(1.34)	0.37(0.29)	0.31(0.24)	0.90(0.71)	1.26(0.99)	1.05(0.83)	3.05(2.40)
	⩾60years	0.56(0.43)	0.48(0.37)	1.58(1.23)	0.29(0.23)	0.25(0.20)	0.83(0.65)	0.99(0.78)	0.85(0.67)	2.80(2.20)
	Total population	0.65(0.50)	0.54(0.43)	1.73(1.35)	0.34(0.27)	0.29(0.22)	0.91(0.71)	1.14(0.90)	0.97(0.76)	3.06(2.41)
URHR	3-6years	1.06(0.81)	1.08(0.82)	1.66(1.26)	0.77(0.56)	0.79(0.57)	1.21(0.87)	1.30(1.05)	1.33(1.07)	2.04(1.64)
	7-12years	0.71(0.54)	0.73(0.56)	1.48(1.13)	0.52(0.38)	0.54(0.39)	1.08(0.78)	0.88(0.71)	0.90(0.72)	1.83(1.47)
	13-17years	0.59(0.45)	0.57(0.44)	0.94(0.72)	0.43(0.31)	0.42(0.30)	0.69(0.49)	0.73(0.59)	0.70(0.57)	1.16(0.93)
	18-59years	0.55(0.42)	0.51(0.39)	1.01(0.77)	0.40(0.29)	0.37(0.27)	0.74(0.53)	0.67(0.54)	0.63(0.51)	1.25(1.00)
	⩾60years	0.50(0.38)	0.50(0.38)	0.92(0.70)	0.37(0.26)	0.37(0.26)	0.67(0.48)	0.62(0.50)	0.62(0.49)	1.13(0.91)
	Total population	0.56(0.43)	0.52(0.40)	1.06(0.81)	0.41(0.29)	0.38(0.28)	0.78(0.56)	0.69(0.55)	0.65(0.52)	1.31(1.05)
MRHR	3-6years	0.58(0.45)	0.55(0.43)	1.15(0.89)	2.13(1.63)	2.03(1.55)	4.24(3.25)	0.80(0.65)	0.76(0.61)	1.60(1.28)
	7-12years	0.34(0.26)	0.35(0.27)	0.66(0.51)	1.25(0.95)	1.28(0.98)	2.46(1.88)	0.47(0.38)	0.48(0.39)	0.92(0.74)

	13-17years	0.25(0.19)	0.24(0.19)	0.47(0.37)	0.92(0.70)	0.90(0.69)	1.76(1.35)	0.34(0.28)	0.34(0.27)	0.66(0.53)
	18-59years	0.29(0.23)	0.30(0.23)	0.53(0.41)	1.08(0.83)	1.10(0.84)	1.95(1.49)	0.41(0.33)	0.41(0.33)	0.73(0.59)
	⩾60years	0.27(0.21)	0.27(0.21)	0.52(0.41)	0.99(0.76)	1.01(0.77)	1.94(1.48)	0.37(0.30)	0.38(0.31)	0.73(0.59)
	Total population	0.29(0.22)	0.29(0.22)	0.55(0.43)	1.06(0.81)	1.06(0.81)	2.04(1.56)	0.40(0.32)	0.40(0.32)	0.77(0.62)
LRHR	3-6years	0.25(0.16)	0.23(0.15)	0.55(0.36)	1.01(0.83)	0.92(0.77)	2.25(1.87)	0.38(0.27)	0.35(0.25)	0.85(0.61)
	7-12years	0.18(0.11)	0.15(0.10)	0.40(0.26)	0.71(0.59)	0.63(0.52)	1.61(1.34)	0.27(0.19)	0.24(0.17)	0.61(0.44)
	13-17years	0.14(0.09)	0.13(0.08)	0.31(0.20)	0.57(0.47)	0.52(0.43)	1.26(1.04)	0.21(0.15)	0.20(0.14)	0.47(0.34)
	18-59years	0.13(0.08)	0.11(0.07)	0.30(0.19)	0.52(0.43)	0.45(0.38)	1.23(1.02)	0.19(0.14)	0.17(0.12)	0.46(0.33)
	⩾60years	0.13(0.08)	0.12(0.08)	0.29(0.18)	0.51(0.42)	0.48(0.40)	1.16(0.96)	0.19(0.14)	0.18(0.13)	0.44(0.32)
	Total population	0.13(0.08)	0.11(0.07)	0.32(0.20)	0.53(0.44)	0.46(0.38)	1.28(1.07)	0.20(0.15)	0.17(0.13)	0.49(0.35)

Table S2. Hazard quotient(mean, median, P95) of NIV of different age group in six river basins in 2022-2024

River basin	Age group	HQ (NIV)								
		**2022**			**2023**			**2024**		
		Mean	Median	P95	Mean	Median	P95	Mean	Median	P95
HRB	3-6years	-[a]	-	-	-	-	-	-	-	-
	7-12years	-	-	-	-	-	-	-	-	-
	13-17years	-	-	-	-	-	-	-	-	-
	18-59years	-	-	-	-	-	-	-	-	-
	≥60years	-	-	-	-	-	-	-	-	-
	Total population	-	-	-	-	-	-	-	-	-
YRB	3-6years	-	-	-	-	-	-	-	-	-
	7-12years	-	-	-	-	-	-	-	-	-
	13-17years	-	-	-	-	-	-	-	-	-

	18-59years	-	-	-	-	-	-	-	-	-
	≥60years	-	-	-	-	-	-	-	-	-
	Total population	-	-	-	-	-	-	-	-	-
MLRY	3-6years	0.042	0.043	0.091	0.027	0.027	0.058	0.192	0.192	0.409
	7-12years	0.026	0.025	0.059	0.017	0.016	0.038	0.117	0.111	0.268
	13-17years	0.017	0.017	0.043	0.011	0.011	0.027	0.078	0.075	0.193
	18-59years	0.020	0.017	0.049	0.013	0.011	0.031	0.091	0.076	0.220
	≥60years	0.016	0.014	0.045	0.010	0.009	0.029	0.071	0.061	0.202
	Total population	0.018	0.015	0.049	0.012	0.010	0.031	0.082	0.070	0.221
URHR	3-6years	0.055	0.056	0.085	-	-	-	0.064	0.065	0.100
	7-12years	0.037	0.038	0.076	-	-	-	0.043	0.044	0.089
	13-17years	0.031	0.029	0.048	-	-	-	0.036	0.034	0.057
	18-59years	0.028	0.026	0.052	-	-	-	0.033	0.031	0.061
	≥60years	0.026	0.026	0.047	-	-	-	0.030	0.030	0.055
	Total population	0.029	0.027	0.055	-	-	-	0.034	0.032	0.064
MRHR	3-6years	0.049	0.047	0.097	0.010	0.009	0.019	0.120	0.114	0.238
	7-12years	0.029	0.029	0.056	0.006	0.006	0.011	0.070	0.072	0.138
	13-17years	0.021	0.021	0.040	0.004	0.004	0.008	0.051	0.051	0.098
	18-59years	0.025	0.025	0.045	0.005	0.005	0.009	0.060	0.062	0.109
	≥60years	0.023	0.023	0.044	0.004	0.005	0.009	0.056	0.057	0.109
	Total population	0.024	0.024	0.047	0.005	0.005	0.009	0.060	0.060	0.115
LRHR	3-6years	-	-	-	0.010	0.010	0.023	0.010	0.009	0.022
	7-12years	-	-	-	0.007	0.007	0.017	0.007	0.006	0.016
	13-17years	-	-	-	0.006	0.005	0.013	0.006	0.005	0.012
	18-59years	-	-	-	0.005	0.005	0.013	0.005	0.004	0.012

≥60years	-	-	-	0.005	0.005	0.012	0.005	0.005	0.011
Total population	-	-	-	0.006	0.005	0.013	0.005	0.004	0.013

[a] Not available

Table S3. The LOD and LOQ values for B-TCTs

Mycotoxins	LOD (μg/kg)	LOQ (μg/kg)
DON	13.3	40
DON-3G	13.3	40
3-AcDON	13.3	40
15-AcDON	13.3	40
NIV	33.3	100

Table S4. wheat consumption (mean, median, and P95 in g/day) for each age group in the six river basins.

River basin	3-6years			7-12years			13-17years			18-59years			≥60years			All population		
	mean	P50	P95	mean	P50	P95	mean	P50	P95	mean	P50	P95	mean	P50	P95	mean	P50	P95
Haihe river basin	166.58	150.00	353.97	225.39	219.67	400.33	258.63	241.67	519.28	296.22	280.00	561.87	306.32	290.67	581.42	283.29	268.67	550.33
Yellow river basin	115.01	110.00	256.67	150.77	140.00	304.67	192.76	178.33	392.75	194.12	166.67	450.00	192.66	166.67	438.75	186.22	160.00	441.67
Yangtze river basin	99.76	100.00	212.98	116.53	110.00	266.33	121.78	116.67	301.20	167.69	140.00	406.67	125.13	107.67	355.33	138.21	116.67	370.00
Upper reaches of Huaihe river	172.53	175.67	270.50	222.46	228.33	462.17	290.19	280.00	460.00	317.13	296.67	586.67	277.70	275.83	505.57	293.48	275.00	558.67
Middle reaches of Huaihe river	191.51	182.00	380.80	213.61	220.00	420.83	246.51	243.33	473.00	343.74	350.00	620.47	300.35	306.67	587.47	306.51	307.00	589.92

Lower reaches of Huaihe river	145.02	132.92	323.95	196.31	173.67	443.75	245.26	226.08	543.33	263.79	232.67	628.27	248.82	232.67	566.67	247.32	213.33	595.67

Text S1: Explanatory notes on the division of the Huaihe River Basin: The Huaihe River Basin can be divided into five secondary water resource zones based on regional distribution characteristics: the upper reaches of Huaihe River (above Wangjiaba), the middle reaches of Huaihe River (from Wangjiaba to the outlet of Hongze Lake), the lower reaches of Huaihe River (below the outlet of Hongze Lake), the Yishushi River Basin, and the coastal rivers of the Shandong Peninsula. The lower reaches of Huaihe River (below the outlet of Hongze Lake), Yishushi River Basin, and Coastal Rivers of Shandong Peninsula into a single study basin, referred to as the Lower Huai River Basin. Additionally, the Huaihe River basin features a large volume of wheat samples, complex and variable weather systems, significant interannual precipitation variability, and highly uneven annual distribution of rainfall. Therefore, based on the regional distribution of water resources, three study areas were established. The locations and boundaries of each secondary zone within the Huaihe River Basin refer to the basin's official website: https://www.hrc.gov.cn/. The locations and boundaries of other basins can be found on their respective basin water conservancy bureau websites (https://www.hwcc.gov.cn/; https://www.cjw.gov.cn/; https://www.yrcc.gov.cn/).

References

1. EFSA Panel on Contaminants in the Food Chain (CONTAM); Knutsen, H.K.; Alexander, J.; Barregård, L.; Bignami, M.; Brüschweiler, B.; Ceccatelli, S.; Cottrill, B.; Dinovi, M.; Grasl-Kraupp, B.; et al. Risks to Human and Animal Health Related to the Presence of Deoxynivalenol and Its Acetylated and Modified Forms in Food and Feed. *EFSA J.* **2017**, *15*, doi:10.2903/j.efsa.2017.4718.
2. Bryła, M.; Ksieniewicz-Woźniak, E.; Yoshinari, T.; Waśkiewicz, A.; Szymczyk, K. Contamination of Wheat Cultivated in Various Regions of Poland during 2017 and 2018 Agricultural Seasons with Selected Trichothecenes and Their Modified Forms. *Toxins* **2019**, *11*, 88, doi:10.3390/toxins11020088.
3. Janssen, E.M.; Sprong, R.C.; Wester, P.W.; De Boevre, M.; Mengelers, M.J.B. Risk Assessment of Chronic Dietary Exposure to the Conjugated Mycotoxin Deoxynivalenol-3-β-Glucoside in the Dutch Population. *World Mycotoxin J.* **2015**, *8*, 561–572, doi:10.3920/WMJ2014.1876.

4. Palacios, S.A.; Erazo, J.G.; Ciasca, B.; Lattanzio, V.M.T.; Reynoso, M.M.; Farnochi, M.C.; Torres, A.M. Occurrence of Deoxynivalenol and Deoxynivalenol-3-Glucoside in Durum Wheat from Argentina. *Food Chem.* **2017**, *230*, 728–734, doi:10.1016/j.foodchem.2017.03.085.
5. Gab-Allah, M.A.; Tahoun, I.F.; Yamani, R.N.; Rend, E.A.; Shehata, A.B. Natural Occurrence of Deoxynivalenol, Nivalenol and Deoxynivalenol-3-Glucoside in Cereal-Derived Products from Egypt. *Food Control* **2022**, *137*, 108974, doi:10.1016/j.foodcont.2022.108974.
6. Xu, A.; Yu, S.; Li, Y.; Liu, H.; Yan, Z.; Wu, A.; Peng, S.; Liu, N. Total Deoxynivalenol Contamination of Wheat Products and Coarse Grains in Shanghai, China: Occurrence and Health Risk Assessment. *Foods* **2024**, *13*, 3373, doi:10.3390/foods13213373.
7. Dong, F.; Wang, S.; Yu, M.; Sun, Y.; Xu, J.; Shi, J. Natural Occurrence of Deoxynivalenol and Deoxynivalenol-3-Glucoside in Various Wheat Cultivars Grown in Jiangsu Province, China. *World Mycotoxin J.* **2017**, *10*, 285–294, doi:10.3920/WMJ2016.2158.
8. Li, F.Q.; Wang, W.; Ma, J.J.; Yu, C.C.; Lin, X.H.; Yan, W.X. Natural Occurrence of Masked Deoxynivalenol in Chinese Wheat and Wheat-Based Products during 2008-2011. *World Mycotoxin J.* **2012**, *5*, 221–230, doi:10.3920/WMJ2012.1412.
